# S12-4 Feasibility evaluation of the Move Well, Feel Good movement behaviours intervention

**DOI:** 10.1093/eurpub/ckad133.061

**Published:** 2023-09-11

**Authors:** Stuart J Fairclough, Lauren Clifford, Lawrence Foweather, Zoe Knowles, Lynne M Boddy, Emma Ashworth, Richard Tyler

**Affiliations:** Movement Behaviours, Nutrition, Health & Wellbeing Research Group, Dept. Sport & Physical Activity, Edge Hill University, UK; Movement Behaviours, Nutrition, Health & Wellbeing Research Group, Dept. Sport & Physical Activity, Edge Hill University, UK; Physical Activity Exchange, Research Institute for Sport & Exercise Sciences, Liverpool John Moores University, UK; Physical Activity Exchange, Research Institute for Sport & Exercise Sciences, Liverpool John Moores University, UK; Physical Activity Exchange, Research Institute for Sport & Exercise Sciences, Liverpool John Moores University, UK; School of Psychology, Liverpool John Moores University, UK; Movement Behaviours, Nutrition, Health & Wellbeing Research Group, Dept. Sport & Physical Activity, Edge Hill University, UK

## Abstract

**Purpose:**

Improving children’s motor competence through school-based physical activity interventions may be a mechanism for promoting positive mental health through psychosocial factors. Move Well, Feel Good (MWFG) is a such an intervention that was co-designed with children, class teachers, physical education (PE) teachers, and school leaders. This presentation describes the feasibility evaluation of MWFG.

**Methods:**

Five primary schools were recruited from a low socioeconomic status community in northwest England. A 10-week intervention was co-designed which aimed to enhance children’s mental health through motor competence and psychosocial development. The core delivery of the programme involved one PE lesson per week devoted to development of locomotor, object control, and stability skills. This was supplemented by optional recess and home activities, and by non-PE curricular activities. Teachers were asked to record details of each MWFG session taught, and feasibility was evaluated against pre-defined criteria using surveys, interviews (teachers), and participatory focus groups (children). Pre- and post-intervention assessments of motor competence, mental health, wellbeing, psychosocial factors, and 24-hour movement behaviours were also completed.

**Results:**

The 5 schools represented 83% of the target number of schools and 108 children consented (43% of target). PE and class teachers were recruited in all schools (100% of target). Intervention dose was reflected by 76% of scheduled PE lessons being delivered. Adherence was strong with >85% of children attending ≥75% of lessons. Positive indicators of acceptability were provided by 86% of children, 83% of PE teachers, and 90% of class teachers. Teachers commented on the children’s positive engagement irrespective of ability, the quality of resources provided, and the challenges of competing demands for curriculum time and spaces to deliver MWFG. The data collection methods were deemed acceptable by 91% of children and 80% of class teachers, and children spoke positively about participating in the data collection. Secondary outcome data collection was completed by 87%-100% of children, with a 0%-6% attrition rate at post-intervention. Descriptive analysis revealed favourable changes in motor competence and mental health.

**Conclusions:**

MWFG was deemed to be feasible to develop further into a pilot trial, with improved school and child recruitment strategies warranted.

